# Association between Onodera’s prognostic nutritional Index and ultrasound-measured muscle thickness in amyotrophic lateral sclerosis: a retrospective cross-sectional study

**DOI:** 10.1080/07853890.2025.2578733

**Published:** 2025-11-01

**Authors:** Tianhua Yang, Ying Wang, Xinyi Yan, Yuxuan Qiu, Ting Lin, Jialei Luo, Jiahui Tong, Jiayi Mao, Yunyi Dai, Yuehui Yu, Min Zhao, Gaoyi Yang

**Affiliations:** a The Fourth School of Clinical Medicine, Zhejiang Chinese Medical University, Hangzhou First People’s Hospital, Hangzhou, China; b Department of Ultrasonography, Affiliated Hangzhou First People’s Hospital, School of Medicine, Westlake University, Hangzhou, China; c Division of Health Sciences, Hangzhou Normal University, Hangzhou, China

**Keywords:** ALS, nutrition, muscle thickness, ultrasound

## Abstract

**Background:**

Amyotrophic lateral sclerosis (ALS) causes progressive muscle wasting. Ultrasound-measured thickness captures this loss. Nutritional status influences ALS prognosis, yet the link between Onodera’s Prognostic Nutritional Index (OPNI) and muscle thickness is unclear.

**Objective:**

To assess whether OPNI correlates with thickness of the first dorsal interosseous (FDI), biceps brachii (BB) and masseter (MM) muscles, and to judge OPNI’s utility as a practical nutrition marker in ALS.

**Methods:**

In this retrospective study of 150 ALS patients, ultrasound quantified FDI, BB and MM thickness. Patients were stratified by OPNI quartile. Group differences were tested with ANOVA/Kruskal–Wallis. Multivariable generalized linear models, adjusting for age, sex, ALSFRS-R, King’s stage, comorbidities and lifestyle factors, examined independent associations; restricted cubic splines probed non-linearity.

**Results:**

Muscle thickness rose progressively across OPNI quartiles (*p* < 0.05 for trend). After full adjustment, each 1-point OPNI increase predicted 0.008 cm thicker FDI (95% CI 0.003–0.014; *p* = 0.004), 0.022 cm thicker BB (95% CI 0.005–0.039; *p* = 0.011) and 0.011 cm thicker MM (95% CI 0.002–0.020; *p* = 0.013). Spline analysis supported a linear relationship.

**Conclusion:**

Higher OPNI independently predicts greater muscle thickness, indicating that better nutritional status parallels reduced muscle wasting in ALS. OPNI’s low cost and rapid availability support its use to flag patients for early nutritional intervention.

## Introduction

Amyotrophic lateral sclerosis (ALS) is a chronic and fatal neurodegenerative disease characterized by the degeneration of upper and lower motor neurons, resulting in muscle weakness and paralysis eventually [1]. Most ALS cases are sporadic, but about 5-10% of cases have a familial genetic background, and the pathogenic genes that have been discovered include SOD1, C9orf72 or TARDBP [2]. Based on clinical manifestations, the site of disease onset varies among patients [3]. ALS progresses continuously, with 50% of patients dying within 30 months of symptom onset [4].

Malnutrition is common in ALS patients [5]. It was observed in patients even without early swallowing problems [6,7]. Fatigue and declining motor function frequently occur alongside weight loss, causing most patients to gradually require assistance with activities of daily living [8,9]. By the time of diagnosis, 50–70% have already experienced weight loss, and its frequency is similar across different sites of onset [10]. A study conducted by the Comprehensive Clinical Research Center at the University of Kentucky Medical Center found that ALS patients experience chronically insufficient energy intake; lean body Mass, muscle strength, and nitrogen balance progressively decrease, while resting energy expenditure increases [11].

Multiple factors contribute to malnutrition and weight loss in ALS. Patients report loss of appetite, which results in reduced caloric intake and worsen ALS outcomes [12]. Other triggers include psychosocial stress and depression leading to anorexia, multiple pharmacological treatments, physical disabilities that limit meal preparation and consumption, and cognitive impairment [13]. Hypermetabolism is another prominent mechanism driving the rapid weight loss observed in ALS patients [14]. Approximately 60% of ALS patients experience increased energy expenditure, which results in decreased fat storage [15,16]. This excess caloric drain arises from a convergence of skeletal-muscle mitochondrial uncoupling, which converts nutrients into heat rather than ATP [17], posterior-hypothalamic atrophy that disrupts central energy homeostasis [18], and systemic inflammatory and lipid-oxidative shifts that sustain a catabolic milieu [19]. In the mouse model of SOD1 mutation, there are early metabolic abnormalities in muscle, which are associated with mitochondrial dysfunction [20]. Similar results have been shown in patients [21]. Equally important, when the disease does involve the bulbar region, either at onset or during progression, weight and nutritional status deteriorate even more rapidly [22]. Recent reviews report that ≥ 80% of people with ALS eventually develop clinically significant dysphagia, and one-third present with bulbar symptoms at diagnosis. Dysphagia leads to reduced caloric intake, aspiration risk that necessitates food-texture or fluid restrictions, and malabsorption from prolonged meal times and early satiety [23,24]. Respiratory-muscle weakness compounds these problems by shortening breath-hold time during swallowing and limiting chewing endurance, further increasing negative energy balance [25]. Nutritional status is a prognostic factor for survival in ALS patients [26,27]. Those with malnutrition exhibited poorer survival, with a 7.7-fold increase in the risk of death [27].

Thus, nutritional status in ALS has a profound influence on both quality of life and survival [28]. Evaluating nutritional status is critically important for managing disease progression in ALS. Currently, Onodera’s Prognostic Nutritional Index (OPNI) has been widely employed to evaluate the nutritional status of patients undergoing various surgeries and those with diverse diseases. A prospective study of 189 gastrointestinal surgery patients showed OPNI accurately quantified surgical risk [29]. In assessing the risk of postoperative complications in Crohn’s disease, an OPNI below 39.8 was identified as an independent risk factor for these complications [30]. Recent research also suggests a relationship between OPNI and prognosis in cancer patients [31,32]. In the neuropsychiatric field, OPNI has also been used to assess the recovery of chronic schizophrenia [33]. Therefore, for ALS patients, OPNI may serve as a simple and objective measure of nutritional status.

Muscle thickness, as an important indicator of muscle health and functional status [34], can directly reflect potential risks of disease progression and declining muscle strength through even subtle changes [35]. Previous studies have shown that ultrasound measurement of muscle thickness demonstrates good reliability and validity for assessing muscle size [34], whereas ultrasound echo intensity is used to evaluate muscle quality [36,37]. Muscle ultrasound has already been applied to diagnose sarcopenia [38]. It also offers excellent repeatability and accessibility, requiring no complex laboratory equipment or conditions, making it highly adaptable for subsequent clinical follow-ups or large-scale population research.

Despite its broad prognostic utility, OPNI has seldom been examined in relation to muscle wasting in ALS. We assessed the association between OPNI and ultrasound-derived thickness of FDI, BB, and MM to clarify how nutritional status influences muscle atrophy in ALS. Establishing this link may sharpen nutritional assessment and guide targeted interventions, ultimately supporting more comprehensive management of ALS.

## Methods

### Study population

This study adopted a retrospective research design. Data were collected from December 2024 to February 2025 at the Motor Neuron Disease Treatment Center of Hangzhou First People’s Hospital, affiliated with the School of Medicine at Westlake University. The following data were retrospectively extracted from the electronic medical record system: baseline demographic characteristics (e.g. gender, age, weight, height, alcohol and smoking history), disease-related information (site of onset, disease duration, ALSFRS-R, King’s Stage and Gene subtypes), nutritional markers (ALB, LYM), and medical history (e.g. hypertension, diabetes). All of them were collected con-currently.

### Inclusion and exclusion criteria

The study population consisted of patients diagnosed with ALS. Inclusion and exclusion criteria are as follows:

#### Inclusion criteria

Diagnosed with ALS by a neurologist, meeting the Gold Coast Criteria

Complete muscle thickness measurements, including ultrasound data for FDI, BB, and MM

Complete nutritional indicator data including ALB and LYM

No severe cognitive or motor impairments that would prevent cooperation with relevant measurements and assessments

#### Exclusion criteria

Presence of any other confirmed muscle disorders

Recent interventions that may affect muscle or nutritional status, such as steroid therapy or high-protein dietary regimens

Severe systemic diseases or comorbidities (e.g. advanced malignancies, severe cardiovascular or pulmonary diseases) that could substantially interfere with the assessment of muscle thickness or nutritional status

Poor image quality or missing data at the time of collection, rendering reliable analysis impossible

## Muscle thickness measurement

Ultrasound assessments were performed using a Samsung R10 system equipped with a 14 MHz broadband linear array transducer. Measurements for each patient were conducted by a randomly assigned, professionally trained ultrasonography doctor. Each muscle was measured three times, with intervals of at least 15 min between measurements to minimize recall bias, and the mean of these three values was used for analysis. All three muscles were assessed in the supine position.

Patients were asked to remain relaxed during scanning to prevent muscle thickening caused by tension or effort. Adequate gel was applied, and the probe was placed gently without additional pressure to avoid external compression effects on muscle thickness. The thickest point of each muscle was identified for repeated measurements. During the ultrasound scanning, we initially measured both sides of each relevant muscle (FDI, BB, and MM). However, to more accurately capture the clinical impact of muscle atrophy and disease progression, only data from the presumed ‘affected side’ which was defined by Neurology Department were included in the final statistical analysis and results. Each patient’s measurements were completed within a single visit to reduce bias. Specific measurement methods for each muscle are as follows,

FDI: With the patient’s palm facing upward, position the thumb at a 30–40° angle relative to the hand’s midline. Measure the FDI thickness in the long-axis view [39].

BB: With the patient’s palm facing upward, the ultrasound probe was placed on the anterior arm at the midpoint between the acromion and the lateral epicondyle, and measurements were taken over the biceps brachii belly parallel to the long axis of the humerus [39].

MM: Keep the patient’s head in a neutral position facing forward, with the mouth naturally closed. Orient the probe parallel to the Masseter fibres and place it at the muscle’s midpoint for measurement [40].

## The OPNI score was calculated as follows

Albumin (ALB) (g/L) + 5 × Lymphocytes (LYM) (×10^9^/L)

## ALSFRS-R and King’s stage

The ALS Functional Rating Scale–Revised (ALSFRS-R) was administered face-to-face by well-trained neurologists who had completed standardized training in neuromuscular disease assessment. King’s stage was assigned according to the original milestone-based framework; however, following the modification validated in Chinese cohorts, the diagnostic milestone (original Stage 2 A) and the involvement of a second region (Stage 2B) were merged into a single Stage 2 to better reflect local disease trajectories [41].

**Gene Subtypes:**

Genotype information was obtained from the Department of Neurology clinical genetics records and abstracted into the study database.

## Data analysis

GraphPad Prism (10.1.2), SPSS (29.0.1.0), and R (4.4.1) were used to analyse. Patients were stratified into quartiles based on OPNI levels. D’Agostino and Pearson test was subsequently used to assess the normality of muscle thickness.

Correlations between muscle thickness and OPNI were analysed using Pearson’s correlation for normally distributed data and Spearman’s correlation for non-normally distributed data. Differences in muscle thickness in those quartiles were compared using one-way ANOVA (MM) or the Kruskal–Wallis test (FDI and BB). Generalized linear models (GLMs) were then constructed to further investigate the association between OPNI and muscle thickness. Candidate confounders were screened in univariable analyses: categorical variables with chi square tests, and continuous variables with one-way ANOVA or with the Kruskal–Wallis test. Those variables showing significant associations were initially included in a partially adjusted GLM. Subsequently, all demographic and clinical factors were incorporated into a fully adjusted GLM to comprehensively assess the independent relationship between OPNI and muscle thickness. In addition, restricted cubic spline (RCS) analysis was conducted to explore potential nonlinear associations between OPNI and muscle thickness. Given the distribution in this cohort, with 148 patients without a known pathogenic variant and two with identified variants, we did not perform gene specific subgroup analyses or OPNI by genotype interaction testing because the counts were too small for reliable inference.

A significance threshold of *p* < 0.05 was used for statistical testing.

All data in this study were retrospectively collected. During routine clinical visits, ALS patients underwent ultrasound assessment of muscle thickness in the first dorsal interosseous, biceps brachii, and masseter muscles to characterize muscle atrophy. To limit the immediate influence of hospital-based interventions, all examinations were performed at admission and prior to the initiation of inpatient medications, nutritional therapy, or rehabilitation. These ultrasound examinations were conducted as part of standard clinical care, and the results were subsequently compiled for use in this study.

This study was approved by the Institutional Review Board of the First Affiliated Hospital of Westlake University School of Medicine, which waived the requirement for informed consent due to the retrospective nature of the study (Protocol No.: 2025ZN035-1). All procedures involving human participants were conducted in accordance with the ethical standards of the institutional and/or national research committee, as well as the 1964 Declaration of Helsinki and its later amendments or comparable ethical standards.

## Results

### Study population

**Figure 1.**
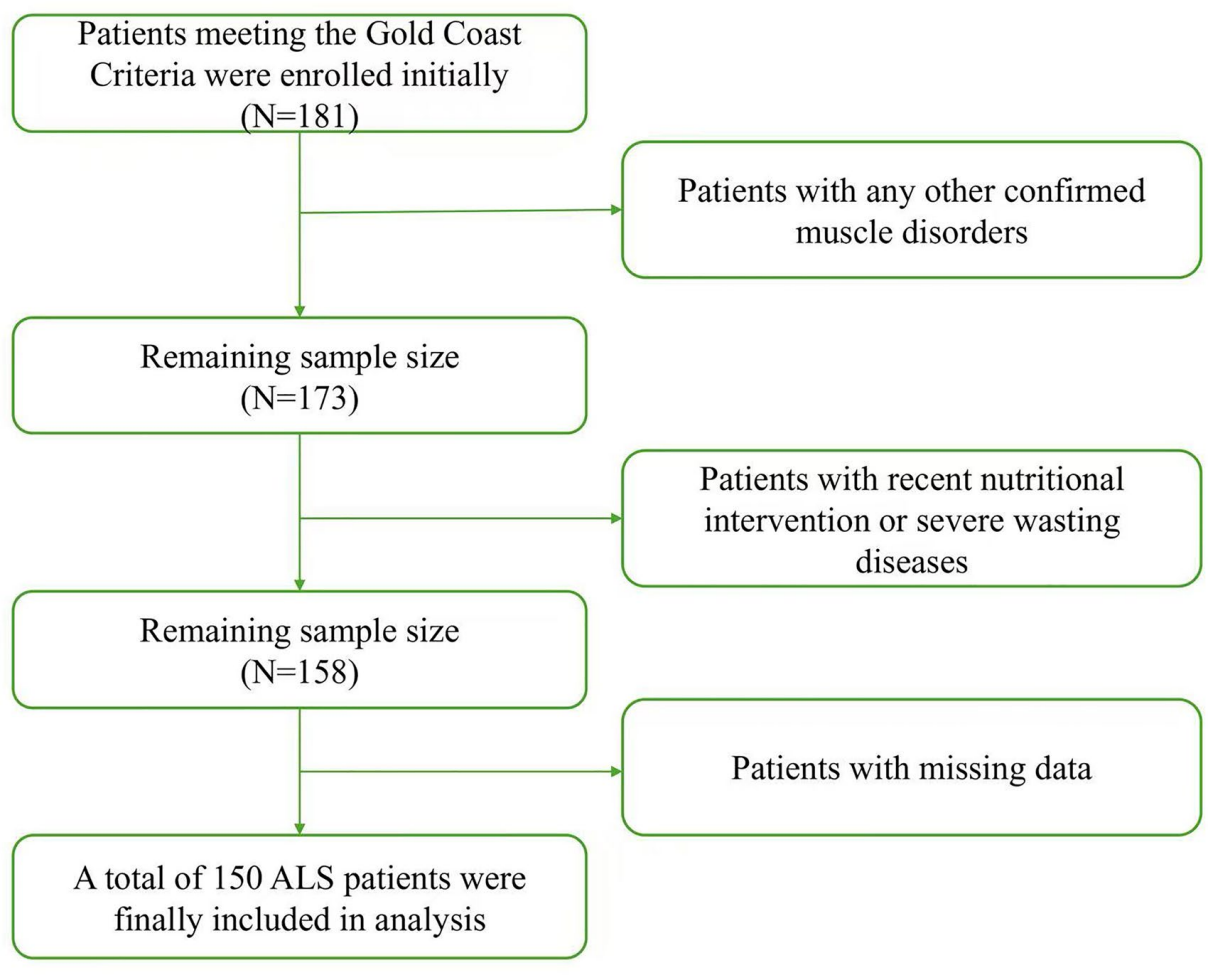
Flowchart of this study.

A total of 181 ALS patients were initially enrolled. Based on the inclusion and exclusion criteria, the final sample sizes were 150, as detailed in Figure 1.

### Correlation analysis

**Figure 2.**
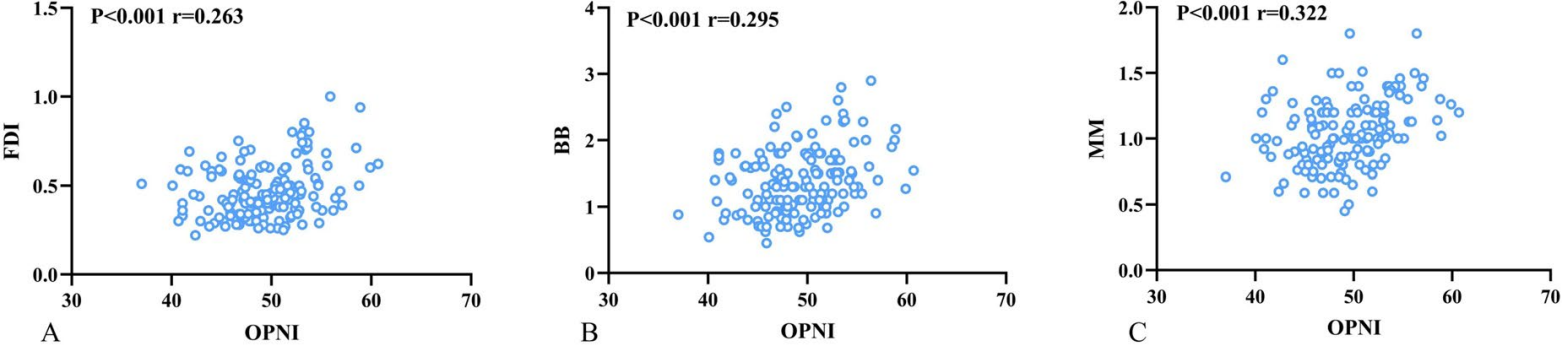
Correlation between OPNI and Muscle Thickness. Note: Scatter plots illustrate the correlation between OPNI and ultrasound-measured thickness of the (A) FDI, (B) BB, and (C) MM. Pearson *r* values and P-values are indicated in each panel. OPNI: Onodera’s Prognostic Nutritional Index; FDI: the first dorsal interosseous; BB: biceps brachii; MM: masseter muscle

OPNI was positively correlated with muscle thickness across all three muscles (All *p* < 0.001; FDI, *r* = 0.263; BB, *r* = 0.295; MM, *r* = 0.322), as shown in Figure 2. Collectively, these correlation analyses underscore the significant relationship between nutritional status and muscle integrity in ALS.

## Baseline information, muscle thickness and clinical variables by OPNI quartiles

**Figure 3.**
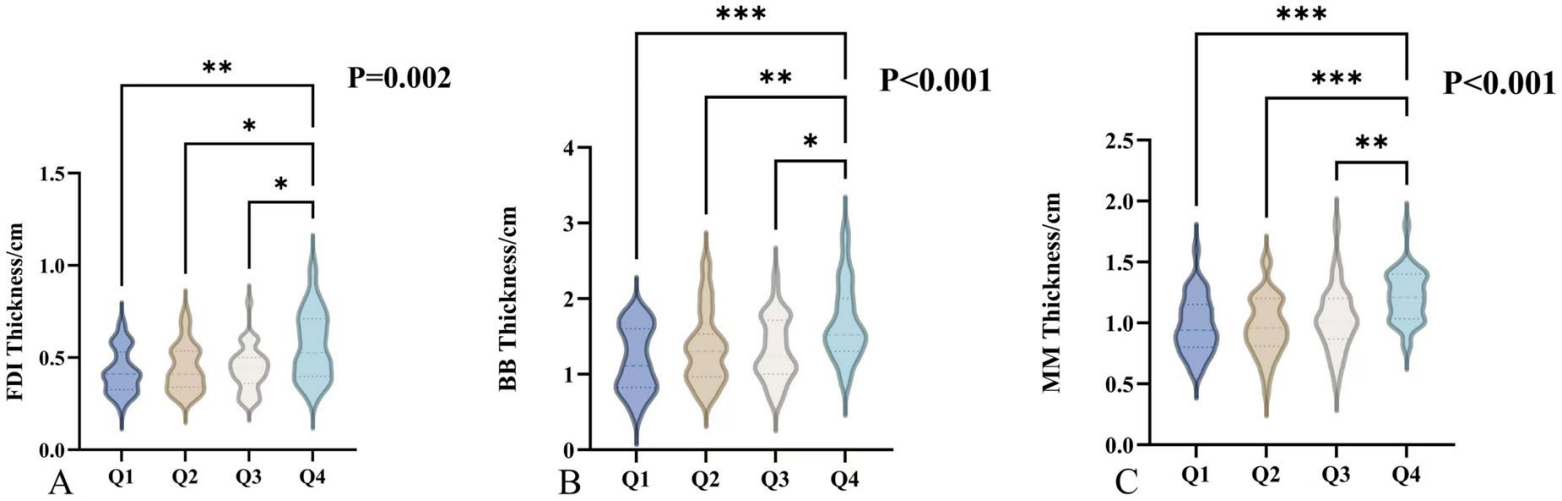
Muscle thickness across OPNI quartiles. Note: Violin plots illustrating the differences in muscle thickness across quartiles (Q1 to Q4) for FDI (A), BB (B), and MM (C). Statistical significance is indicated by asterisks (*P< 0.05, **P < 0.01, ***P < 0.001). OPNI: Onodera’s Prognostic Nutritional Index; FDI: the first dorsal interosseous; BB: biceps brachii; MM: masseter muscle

Table 1 shows that across ascending OPNI quartiles (Q1 ≤ 46.5, Q2 46.5 to 49.2, Q3 49.2 to 52.4, Q4 ≥ 52.4; *N* = 37, 37, 38, 38), median FDI thickness increased from 0.41 cm (0.33 to 0.51) in Q1 to 0.53 cm (0.41 to 0.71) in Q4 (*p* = 0.002). Median BB thickness rose from 1.11 cm (0.85 to 1.60) to 1.52 cm (1.30 to 2.00) (*p* < 0.001). Mean MM thickness increased from 0.98 ± 0.23 cm to 1.22 ± 0.20 cm (*p* < 0.001). Patients in higher OPNI quartiles were younger, with median age decreasing from 55.00 years (50.00 to 62.00) to 49.00 years (41.25 to 56.00) (*p* = 0.014), and had higher ALSFRS R scores, rising from 23.00 (15.00 to 27.00) to 32.00 (26.00 to 37.00) (*p* < 0.001). The proportion of males increased from 51.4% to 86.8% (*p* = 0.002). King’s stage distribution differed across quartiles (*p* = 0.015), with stage 1 increasing from 5.4% to 18.4% and stage 4B decreasing from 45.9% to 15.8%. Disease duration, site of onset, hypertension, diabetes, smoking, alcohol use, and gene subtype distribution did not differ significantly among quartiles (all *p* > 0.05). Muscle thickness significantly differed among the quartiles for all three muscles (FDI, *p* = 0.002; BB, *p* < 0.001; MM, *p* < 0.001), as detailed showed in Figure 3.

**Table 1.** Baseline profiles and muscle thickness across OPNI categories.

	Q1 ≤ 46.5	Q2:46.5—49.2	Q3:49.2—52.4	Q4 ≥ 52.4	
Variables	*N* = 37	*N* = 37	*N* = 38	*N* = 38	*P-Value*
**FDI Thickness/cm**	0.41(0.33, 0.51)	0.41(0.34, 0.54)	0.44(0.36, 0.50)	0.53(0.41, 0.71)	0.002**
**BB Thickness/cm**	1.11(0.85, 1.60)	1.30(0.97, 1.53)	1.30(1.00, 1.72)	1.52(1.30, 2.00)	<0.001***
**MM Thickness/cm**	0.98 ± 0.23	0.97 ± 0.24	1.03 ± 0.26	1.22 ± 0.20	<0.001***
**Age**	55.00 (50.00,62.00)	53.00 (47.00,58.00)	49.50 (42.25,58.75)	49.00 (41.25,56.00)	0.014*
**ALSFRS-R**	23.00 (15.00,27.00)	21.00 (18.00,27.00)	23.50 (17.25,31.00)	32.00 (26.00,37.00)	<0.001***
**Gender**					0.002**
Male	19(51.4%)	18(48.6%)	25(65.8%)	33(86.8%)	
Female	18(48.6%)	19(51.4%)	13(34.2%)	5(13.2%)	
**Disease Duration/Month.**	24.00	24.00 (12.00,36.00)	24.00 (12.00,36.00)	24.00 (12.00,34.50)	0.365
	(12.00,42.00)				
**Site of Onset**					0.457
Spinal-Onset	30(81.1%)	34(91.9%)	35(92.1%)	33(86.8%)	
Bulbar-Onset	6(16.2%)	2(5.4%)	1(2.6%)	4(10.5%)	
Others	1(2.7%)	1(2.7%)	2(5.3%)	1(2.6%)	
**Hypertension**					0.503
Yes	17(45.9%)	14(37.8%)	15(39.5%)	11(28.9%)	
No	20(54.1%)	23(62.2%)	23(60.5%)	27(71.1%)	
**Diabetes**					0.275
Yes	7(18.9%)	2(5.4%)	3(7.9%)	3(7.9%)	
No	30(81.1%)	35(94.6%)	35(92.1%)	35(92.1%)	
**Smoking**					0.261
Yes	2(5.4%)	1(2.7%)	5(13.2%)	5(13.2%)	
No	35(94.6%)	36(97.3)	33(86.8%)	33(86.8%)	
**Alcohol**					0.149
Yes	2(5.4%)	0(0.0%)	3(7.9%)	0(0.0%)	
No	35(94.6%)	37(100.0%)	35(92.1%)	38(100.0%)	
**King’s Stage**					0.015**
1	2(5.4%)	3(8.1%)	0(0.0%)	7(18.4%)	
2	10(27.0%)	10(27.0%)	10(26.3%)	15(39.5%)	
3	7(18.9%)	13(35.1%)	17(44.7%)	10(26.3%)	
4A	1(2.7%)	2(5.4%)	3(7.9%)	0(0.0%)	
4B	17(45.9%)	9(24.3%)	8(21.1%)	6(15.8%)	
**Gene Subtypes**					1.000
Sporadic	37 (100.0%)	37 (100.0%)	37 (97.4%)	37 (97.4%)	
FUS	0(0.0%)	0(0.0%)	1 (2.6%)	0(0.0%)	
TARDBP	0(0.0%)	0(0.0%)	0(0.0%)	1 (2.6%)	

FDI: The First Dorsal Interosseous; BB: Biceps Brachii; MM: Masseter Muscle; AL%FRS-R: ALS Functional Rating Scale-Revised.

Statistical significance is indicated as follows: *p < 0.05, **p < 0.01, ***p < 0.001.

## Association between OPNI and muscle thickness

The association between OPNI and muscle thickness is shown in Table 2, using GLM. Because ALSFRS-R and King’s stage largely overlap in clinical meaning and were strongly correlated in our data with ρ= −0.726 (Supplementary Figure 1), we did not include them together to avoid redundancy and retained King’s stage as the primary severity covariate. Considering the potential impacts of general epidemiological factors and all candidate predictors showed acceptable collinearity (VIF < 5; Supplementary Table S1), the final model was adjusted for gene subtypes, gender, age, history of diabetes, history of hypertension, smoking history, alcohol history, site of onset, King’s stage and disease duration. In model 2, higher OPNI was associated with greater muscle thickness. Each 1 unit increase in OPNI corresponded to an increase of 0.008 cm in FDI thickness (95% CI 0.003 to 0.014, *p* = 0.004), 0.022 cm in BB thickness (95% CI 0.005 to 0.039, *p* = 0.011), and 0.011 cm in MM thickness (95% CI 0.002 to 0.020, *p* = 0.013). When OPNI was categorized into quartiles, participants in Q1 to Q3 had significantly lower thickness than Q4 across all muscles. For FDI, regression coefficients for Q1 to Q3 versus Q4 ranged from −0.121 to −0.100 with 95% confidence intervals spanning −0.191 to −0.034 and P values up to 0.003. For BB, coefficients ranged from −0.348 to −0.248 with confidence intervals spanning −0.567 to −0.040 and P values from 0.002 to 0.019. For MM, coefficients ranged from −0.183 to −0.157 with confidence intervals spanning −0.288 to −0.045 and P values from <0.001 to 0.006. Ordered trend tests were significant for all three sites, with P for trend equal to 0.003 for FDI, 0.004 for BB, and 0.008 for MM.

**Table 2.** Association between OPNI and muscle thickness.

		Unadjusted Model			Model 1 ^a^			Model 2 ^b^		
	OPNI	B	95%CI	*P-Value*	B	95%CI	*P-Value*	B	95%CI	*P-Value*
FDI	Q1	−0.285	−0.415— −0.155	<0.001	−0.133	−0.439— −0.156	<0.001	−0.121	−0.191— −0.051	<0.001
	Q2	−0.242	−0.372— −0.111	<0.001	−0.118	−0.375— −0.095	<0.001	−0.100	−0.165— −0.034	0.003
	Q3	−0.254	−0.383— −0.124	<0.001	−0.124	−0.367— −0.096	<0.001	−0.109	−0.176— −0.043	0.001
	Q4	Ref.			Ref.			Ref.		
	Continuous	0.020	0.010—0.031	<0.001	0.009	0.003—0.015	0.002	0.008	0.003—0.014	0.004
	P for Trend	0.086	0.044—0.127	<0.001	0.039	0.016— 0.062	0.001	0.034	0.012— 0.057	0.003
BB	Q1	−0.342	−0.491— −0.194	<0.001	−0.365	−0.582— −0.147	0.001	−0.348	−0.567— −0.129	0.002
	Q2	−0.228	−0.377— −0.080	0.003	−0.269	−0.478— −0.061	0.011	−0.259	−0.464— −0.053	0.014
	Q3	−0.237	−0.385— −0.089	0.002	−0.250	−0.455— −0.045	0.017	−0.248	−0.456— −0.040	0.019
	Q4	Ref.			Ref.			Ref.		
	Continuous	0.025	0.012— 0.037	<0.001	0.024	0.006— 0.042	0.008	0.022	0.005— 0.039	0.011
	P for Trend	0.103	0.056— 0.150	<0.001	0.110	0.040— 0.179	0.002	0.103	0.034— 0.172	0.004
MM	Q1	−0.235	−0.340— −0.130	<0.001	−0.127	−0.237— −0.016	0.025	−0.157	−0.268— −0.045	0.006
	Q2	−0.245	−0.350— −0.140	<0.001	−0.156	−0.262— −0.050	0.004	−0.183	−0.288— −0.078	<0.001
	Q3	−0.189	−0.293— −0.085	<0.001	−0.115	−0.219— −0.011	0.031	−0.159	−0.265— −0.052	0.003
	Q4	Ref.			Ref.			Ref.		
	Continuous	0.019	0.010— 0.027	<0.001	0.010	0.001— 0.019	0.025	0.011	0.002— 0.020	0.013
	P for Trend	0.076	0.042— 0.110	<0.001	0.041	0.006— 0.077	0.023	0.048	0.012— 0.084	0.008

OPNI was included in the Generalized Linear Model (GLM) as both a categorical and a continuous variable.

FDI: The First Dorsal Interosseous BB: Biceps Brachii MM: Masseter Muscle.

^a^ : Model 1: adjusted for gender, age and King’s Stage.

^b^ : Model 2: gender, age, history of diabetes, history of hypertension, smoking history, alcohol history, site of onset, disease duration, King’s Stage and gene subtypes.

## Restricted cubic spline (RCS) curves depicting association between OPNI and muscle thickness

**Figure 4.**
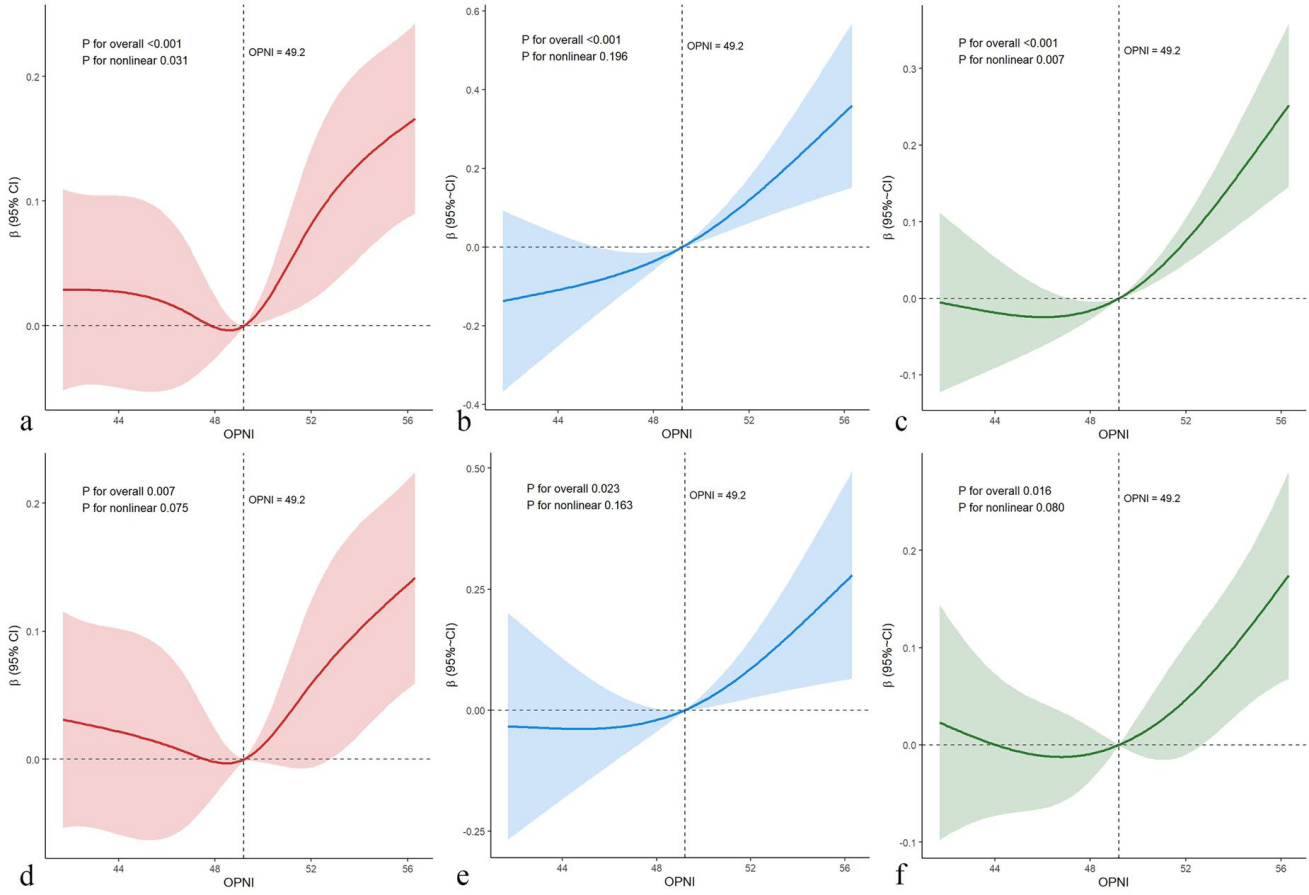
Restricted Cubic Spline Analysis Results for the Relationship Between Different Muscle Thicknesses and OPNI. Note: a–c: RCS analyses of OPNI with FDI, BB, and MM in unadjusted models. d–f: corresponding multivariable adjusted models. Curves display β, and the shaded areas indicate 95% confidence intervals. The vertical dashed line marks OPNI = 49.2. The horizontal dashed line marks β = 0. Each panel reports the model-based P for the overall association and for the nonlinear component. OPNI: Onodera’s Prognostic Nutritional Index; FDI: the first dorsal interosseous; BB: biceps brachii; MM: masseter muscle

Restricted cubic spline models demonstrated a positive association between OPNI and ultrasound-measured muscle thickness. In the unadjusted analyses (Figure 4a–c), the overall association was significant for all muscles (FDI, BB, and MM; all P for overall <0.001). A nonlinear component was evident for the first dorsal interosseous (P for nonlinearity = 0.031) and the masseter (P for nonlinearity = 0.007), but not for the biceps brachii (P for nonlinearity = 0.196). After adjustment (Figure 4d–f), the overall association remained significant for FDI (P for overall = 0.007), BB (P for overall = 0.023), and MM (P for overall = 0.016), whereas the nonlinearity test was not significant for any muscle (FDI P for nonlinearity = 0.075; BB P for nonlinearity = 0.163; MM P for nonlinearity = 0.080). The adjusted exposure–response is best interpreted as approximately linear across the observed OPNI range. Curves are displayed on a relative effect scale as β with 95% confidence intervals and are zero centered at OPNI equals 49.2, the cohort median, which is shown as a vertical reference line to aid orientation rather than as a clinical threshold.

## Discussion

Researches consistently indicate that malnutrition is very closely linked with muscle atrophy and functional decline [42,43]. Weight loss, reduced BMI, and insufficient protein intake are significant factors contributing to decreased muscle mass, which ultimately leads to impaired motor function and reduced survival [44–46]. These observations underscore the crucial role of nutritional status in ALS management [47]. Consistent with these studies, our study demonstrates a clear link between patients’ nutritional status and their skeletal muscle integrity in ALS. Specifically, OPNI was linearly and positively associated with ultrasound-measured muscle thickness across multiple muscle groups. In practical terms, ALS patients with higher OPNI (indicating better immune-nutritional status) tended to have thicker muscles, whereas those with low OPNI had appreciably thinner muscles.

Several pathophysiological mechanisms may explain the observed association between low OPNI and reduced muscle thickness in ALS. First, ALS patients frequently experience malnutrition due to a combination of dysphagia, hypermetabolism, and disuse atrophy [13]. Nearly half of ALS patients are clinically malnourished at the time of diagnosis [48]. This occurs in part due to decreased oral intake, from swallowing difficulties and limb weakness impairing feeding, and in part due to a paradoxical hypermetabolic state. ALS is often characterized by a vicious cycle of under-nutrition and increased resting energy expenditure – essentially, the body’s highest energy-consuming tissues are wasting, yet total energy burn remains abnormally high [17]. This paradox exacerbates caloric deficits and accelerates muscle catabolism. Indeed, hypermetabolic ALS patients have been shown to experience faster functional decline and shorter survival [49]. OPNI, as a composite of serum albumin and lymphocyte count, captures two key aspects of this pathophysiology: poor nutritional intake or reserves and systemic inflammation. Serum albumin in ALS correlates strongly with the inflammatory state rather than just nutritional status [50], and lymphocyte count can serve as an indicator of the patient’s nutritional status rather than just an inflammatory marker as well [51].

Notably, this relationship held true even for the MM that some literature suggests is spared in early ALS [52,53]. However, in our study, we found a significant positive correlation between masseter thickness and the thickness of hand and upper limb muscles. Several factors may explain this finding. First, a substantial proportion of our participants were in middle to late stages of ALS, and prolonged denervation can eventually involve the masseter. Second, malnutrition and hypermetabolism are both common in ALS, driving systemic catabolism that affects skeletal muscles throughout the body, including cranial muscles. In fact, there are some studies suggest that masseter is highly associated with nutrition status in those who do not suffer from ALS [54,55]. Finally, bulbar involvement, especially in bulbar onset or advanced progression [56], impairs chewing and swallowing, worsens energy imbalance, and accelerates muscle wasting. Overall, our results broaden the perspective that even muscles traditionally considered ‘preserved’ may show measurable atrophy when the patient’s overall nutritional and inflammatory status is poor.

Our findings carry several clinical implications. They highlight OPNI as a quick, accessible tool for nutritional screening in ALS that also correlates with objective muscle status. In the clinic, OPNI could be calculated from routine laboratory tests, which are easy to get and used to identify patients at risk of malnutrition-related muscle loss. In a study assessing nutritional status with a tool that is validated in a vastly different population of peri-surgical patients a comparison with height, weight and BMI measurements is critical. But in our data collection process, over 50% of patients arrived by wheelchair or stretcher, making it impossible to obtain accurate height and weight measurements or even to collect these data at all. However, this issue is not confined to very late-stage patients. For example, in King’s stage 1 patients with lower limb involvement only, weakness can already impair independent ambulation, often necessitating the use of a wheelchair or stretcher upon admission, which in turn makes it challenging to obtain accurate anthropometric data. Furthermore, BMI cannot differentiate between fat and muscle mass and may be confounded by factors such as fluid retention or oedema, which can obscure the patient’s true nutritional state [57,58]. Thus, an ALS patient’s OPNI might offer prognostic insight alongside traditional factors like BMI or weight loss rate to identify patients who might benefit from timely nutritional interventions to potentially improve outcomes in a quantitative way.

This study has constraints that should inform interpretation. The cross-sectional, single time-point design prevents establishing temporal ordering or causal direction between nutritional status and muscle thickness, and reverse causation cannot be excluded. Although measurements were obtained at admission to reduce immediate in-hospital treatment effects, residual confounding from prior outpatient medications, nutrition, and rehabilitation remains possible. During enrollment, the need for assisted transport and extended clinic time strained available resources and precluded recruitment of matched non-ALS participants. As a result, no healthy or neurological disease controls were included, limiting external validity and preventing benchmarking of ultrasound thickness and OPNI against reference ranges. Imaging focused on three clinically relevant muscles and primary analyses used the clinically more affected side, a feasible approach in busy clinics but one that may underrepresent lower-limb involvement and bilateral asymmetry that are important in ALS. Genetic heterogeneity may modify phenotypes, yet the sample did not support genotype-stratified analyses or interaction testing. OPNI components are sensitive to inflammation, BMI was unavailable, and inflammatory markers were not paired, leaving potential confounding by body habitus and systemic inflammation. Finally, the 14 MHz probe may miss very small thickness changes compared with higher frequency transducers.

Future work will prioritize prospective longitudinal cohorts to clarify temporal relationships between OPNI and muscle changes and to test whether improvements in nutritional status attenuate atrophy or delay functional decline. We plan to recruit age- and sex-matched healthy controls and neurological disease controls using a harmonized protocol, expand the muscle panel to include lower-limb groups such as rectus femoris and quadriceps, and perform bilateral acquisitions. Acquisition settings will be standardized with centralized quality control, higher frequency probes at or above 18 MHz, and rigorous capture of medication, nutrition, and rehabilitation exposures. We will collect BMI and pair OPNI with inflammatory markers such as C-reactive protein, create reference ranges to enable z-score or percentile interpretation, and implement genotype-aware analyses with prespecified strata and interaction tests. Multicentre enrollment, preregistered analysis plans, and adequately powered interventional studies that target optimization of OPNI may be used to evaluate clinical impact and strengthen generalizability.

## Conclusion

In this ALS cohort, higher OPNI correlated with greater ultrasound-measured muscle thickness in clinically relevant muscles. This aligns with prior studies indicating that better nutritional status relates to more favorable ALS trajectories and that muscle ultrasound captures disease involvement at the bedside. Because the adjusted nonlinearity test was not significant, we interpret the relationship as approximately linear and recommend longitudinal studies using standardized nutrition protocols and bilateral, multi-muscle ultrasound to evaluate prognostic and interventional implications in the future.

## Supplementary Material

Supplementary Table S1.docx

Supplementary Figure 1.jpeg

## Data Availability

The datasets analysed during the current study are not publicly available due to the protection of patient privacy but are available from the corresponding author on reasonable request.
