# Supplementary material for: Association between Onodera’s prognostic nutritional Index and ultrasound-measured muscle thickness in amyotrophic lateral sclerosis: a retrospective cross-sectional study
Source: Ann Med. 2025 Nov 1;57(1):2578733. doi: 10.1080/07853890.2025.2578733 (PMC12581742; doi:10.1080/07853890.2025.2578733)
Supplement: Supplementary Table S1.docx [file IANN_A_2578733_SM9960.docx]

| **Variable** | **B value** | **SE** | **Beta** | **T value** | **P value** | **Tolerance** | **VIF** |
| --- | --- | --- | --- | --- | --- | --- | --- |
| Gene subtypes | 0.240 | 0.102 | 0.183 | 2.356 | 0.020 | 0.965 | 1.036 |
| OPNI | 0.008 | 0.003 | 0.234 | 2.732 | 0.007 | 0.793 | 1.262 |
| Gender | 0.004 | 0.026 | 0.011 | 0.137 | 0.892 | 0.835 | 1.198 |
| Disease duration | -0.001 | 0.000 | -0.118 | -1.511 | 0.133 | 0.958 | 1.044 |
| Site of onset | 0.006 | 0.026 | 0.016 | 0.210 | 0.834 | 0.969 | 1.032 |
| Hypertension | -0.027 | 0.025 | -0.087 | -1.097 | 0.275 | 0.919 | 1.088 |
| Diabetes | 0.067 | 0.041 | 0.133 | 1.617 | 0.108 | 0.862 | 1.160 |
| Smoking | 0.137 | 0.054 | 0.256 | 2.533 | 0.012 | 0.569 | 1.758 |
| Alcohol | -0.150 | 0.082 | -0.179 | -1.837 | 0.068 | 0.615 | 1.627 |
| Age | 0.000 | 0.001 | -0.008 | -0.096 | 0.924 | 0.840 | 1.191 |
| Kings stage | -0.003 | 0.009 | -0.029 | -0.362 | 0.718 | 0.885 | 1.130 |

**Supplementary Table S1. Collinearity diagnostics for the multivariable linear regression model**

Covariates entered in the model were gene subtypes, Onodera’s Prognostic Nutritional Index, gender, disease duration, site of onset, hypertension, diabetes, smoking, alcohol, age, and Kings stage. Columns report the unstandardized coefficient B, standard error SE, standardized coefficient Beta, t statistic, P value, tolerance, and variance inflation factor as collinearity diagnostics.

OPNI Onodera’s Prognostic Nutritional Index; VIF variance inflation factor; SE standard error.
