# Supplementary figures and images for: Association between Onodera’s prognostic nutritional Index and ultrasound-measured muscle thickness in amyotrophic lateral sclerosis: a retrospective cross-sectional study
Source: Ann Med. 2025 Nov 1;57(1):2578733. doi: 10.1080/07853890.2025.2578733 (PMC12581742; doi:10.1080/07853890.2025.2578733)

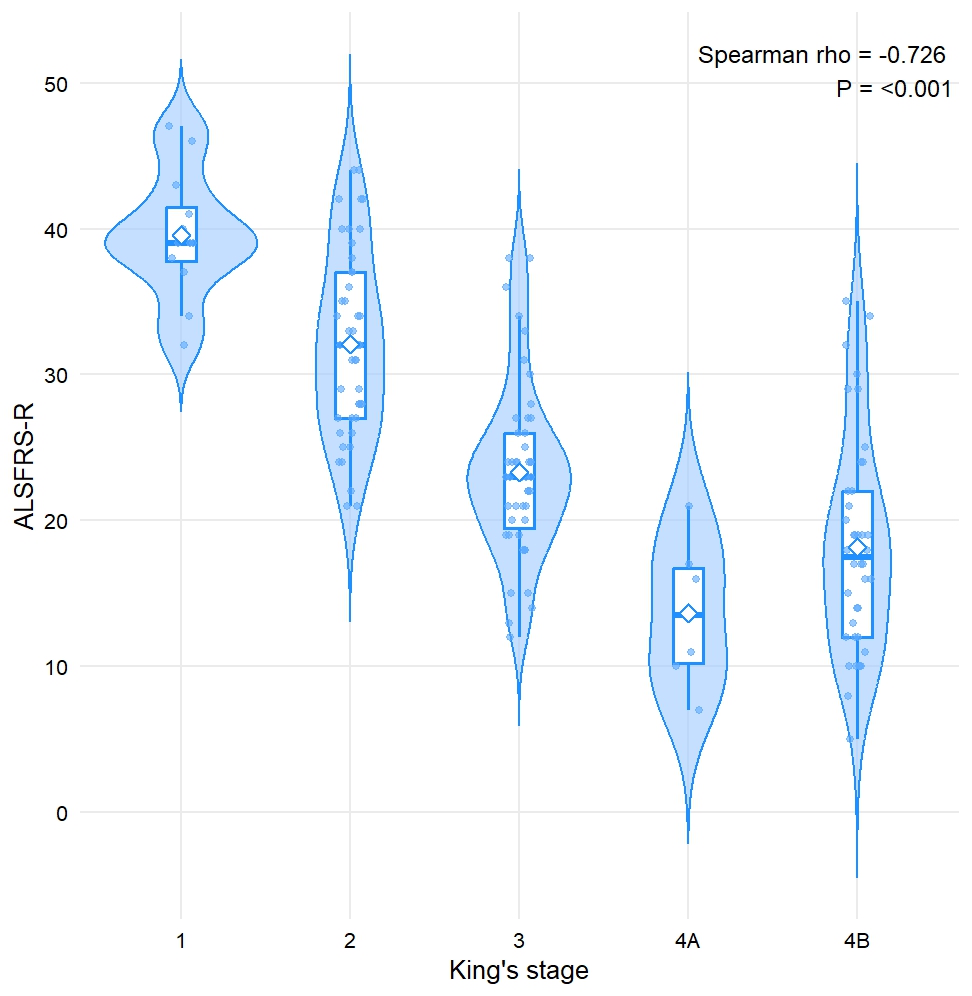

Supplement: Supplementary Figure 1.jpeg [file IANN_A_2578733_SM9959.jpeg]
